# Complete genome of tomato yellow leaf curl Kanchanaburi virus (DNA-A and DNA-B) causing significant yield loss in tomato crops in the Philippines

**DOI:** 10.1128/mra.00257-25

**Published:** 2025-08-06

**Authors:** Elizabeth G. Panerio, Lawrence I. Norte, Jeshua Paul P. Antonino, Madel L. Canceran, Ricci Leighn Divine A. Anque, E-jay G. Dequilla

**Affiliations:** 1 Department of Science and Technology, Industrial Technology Department Institute, Metro Manila, Philippines; Queens College Department of Biology, Queens, New York, USA

**Keywords:** TYLCV, tomato yellow leaf curl virus, tomato

## Abstract

Tomato yellow leaf curl virus is a major threat to tomato production globally. In the Philippines, it poses a significant challenge to farmers. The whole genome of the tomato yellow leaf curl virus Philippine isolate was sequenced to develop molecular markers and aid early detection.

## ANNOUNCEMENT

Tomato yellow leaf curl virus (TYLCV) is a viral plant pathogen of significant economic importance. This virus belongs to the *Begomovirus* genus within the *Geminiviridae* family, which includes viruses transmitted by the whitefly *Bemisia tabaci*. Genomes of geminiviruses are either bipartite, having two DNA components (DNA-A and DNA-B), or monopartite, which has a single DNA component (1). In the Philippines, farmers have recognized TYLCV as a primary agricultural problem affecting tomato production nationwide.

TYLCV was isolated from infected leaf samples from Calcoa Farm, Barangay Cabintan, Ormoc City, Leyte (11° 4′ 50.448″' N, 124° 43′ 22.6992″' E). Viral genomic DNA was extracted using the traditional cetyltrimethylammonium bromide (CTAB) method. Leaves were frozen using liquid nitrogen and were grounded into powder prior to extraction. One hundred milligrams (100 mg) of the sample was placed in a 50 mL falcon tube with 8 mL of CTAB buffer preheated at 60°C. It was incubated at 60°C, centrifuged, and the top phase was collected. Nucleic acids were precipitated with cold isopropanol and incubated at −20°C overnight. The pellet was washed with 70% ethanol, dried, and resuspended in TE buffer.

The genome sequencing library was prepared using the NextSeq 1000 platform P2 300 Cycles, paired-end settings. All bioinformatics tools used were run in default unless otherwise indicated. Raw reads were processed using Trimmomatic v0.36 (2) to trim low-quality (<Q20) and remove adapter sequences (2). Reference-based assembly was carried out using minimap version 2.17 (3) using the -t parameter, and post-alignment processing was done using samtools version 1.3.1 (4).

**Fig 1:**
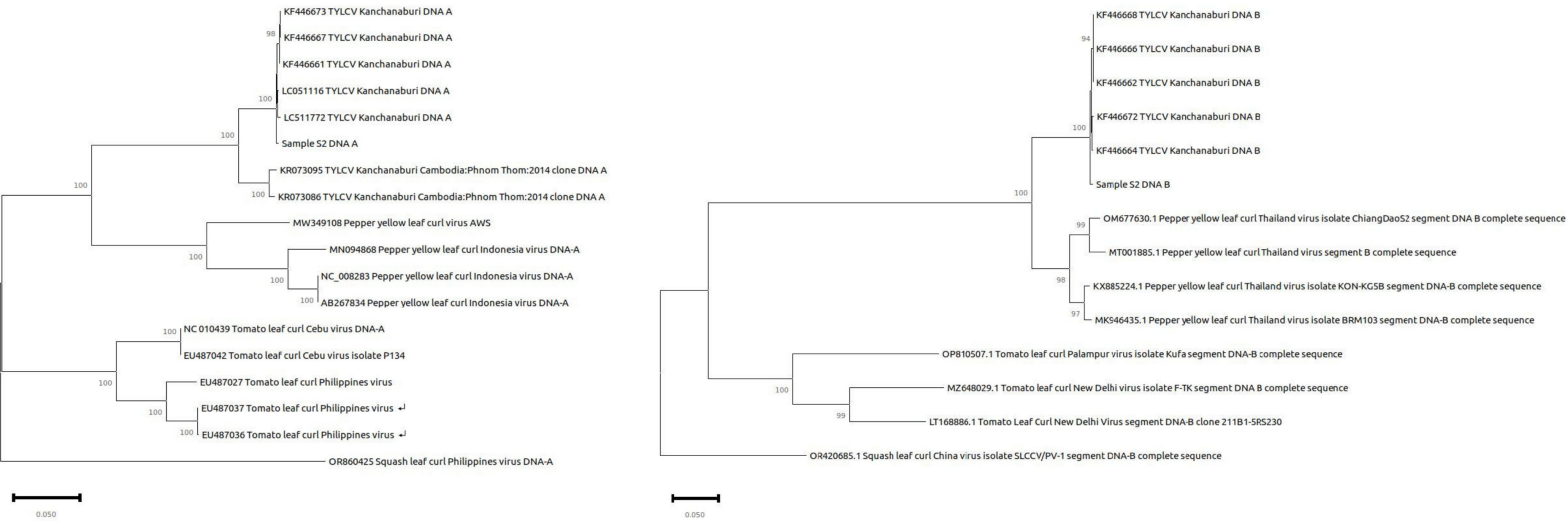
Phylogenetic tree of tomato yellow leaf curl and leaf curl virus DNA-A (left) and DNA-B (right) isolates constructed by the maximum likelihood method. Sample S2 DNA-A and DNA-B is a putative TYLCV Kanchanaburi DNA-A and DNA-B in this analysis. Bootstrap values with >80% are shown at the nodes. An isolate of squash leaf curl Philippines isolate virus DNA-A and DNA-B segment was used as an outgroup.

Sequencing produced 41,573,995 raw reads, and assembly yielded a genome size of 2752 bp, potentially covering the entire viral sequence. The genome has 53% GC and N_50_ of 2,058, comprising three contigs. Taxonomic identification using NCBI—Basic Local Alignment Search Tool—BLAST (5) showed 99.38% identity of TYLCV DNA-A to tomato yellow leaf curl Kanchanaburi virus isolate P4 segment DNA-A, complete sequence (NC_005812.1) and 98.51% identity of TYLCV DNA-B with Tomato yellow leaf curl Kanchanaburi virus isolate P4 segment DNA-B complete sequence (NC_005 811.1) (Table 1), both of which are reference genome, confirming the virus’ identity (Fig. 1). Further confirmation was through computation of average nucleotide identity through ANI Calculator (6) run using the default parameters for ANIb, which showed 100% genome coverage for both DNA segments (A and B) with OrthoANI scores of 97.30% and 97.75%, respectively.

**TABLE 1:** Top species-specific BLAST results for all contigs

Contig name	NCBI reference name	Description	Max score	Total score	Query cover	e value	% identity	Query length
Sample S2 DNA A	NC_005812.1	Tomato yellow leaf curl Kanchanaburi virus isolate P4 segment DNA-A, complete sequence	5003	5003	100	0	99.38	2752
Sample S2 DNA B	NC_005 811.1	Tomato yellow leaf curl Kanchanaburi virus isolate P4 segment DNA-B, complete sequence	4892	4892	100	0	98.51	2752

For annotation, Prokka 1.14.6 (7) was used. Annotation identified six genes, all of which are protein-coding sequences. Further functional annotation using EggNOG (8) showed the TYLCV DNA A’s association with viral genome and replication and host-virus interactions. Geminivirus Rep catalytic domain is the most significant match, suggesting a role in viral DNA replication. This is further supported by Gene Ontology terms related to viral life cycle (GO:0016032), viral genome replication (GO:0019058), and viral DNA synthesis (GO:0039687). On the other hand, TYLCV DNA B’s functional annotation showed its association with the host cell endoplasmic reticulum membrane, suggesting a role in host-virus interactions.

## Data Availability

This genome project has been registered at NCBI under BioProject accession number PRJNA1131566. Raw reads have been deposited at the NCBI Sequence Archive (SRA) under the accession number SRR29730463. The genome sequence of both TYLCV segments A and B has been deposited at GenBank under the accession numbers PP990580.1 (segment A) and PP990579 (segment B).

## References

[1] Ramesh SV, Sahu PP, Prasad M, Praveen S, Pappu HR. 2017. Geminiviruses and plant hosts: a closer examination of the molecular arms race. Viruses 9:256. doi:10.3390/v9090256 28914771 PMC5618022

[2] Bolger AM, Lohse M, Usadel B. 2014. Trimmomatic: a flexible trimmer for Illumina sequence data. Bioinformatics 30:2114–2120. doi:10.1093/bioinformatics/btu170 24695404 PMC4103590

[3] Li H. 2018. Minimap2: pairwise alignment for nucleotide sequences. Bioinformatics 34:3094–3100. doi:10.1093/bioinformatics/bty191 29750242 PMC6137996

[4] Li H, Handsaker B, Wysoker A, Fennell T, Ruan J, Homer N, Marth G, Abecasis G, Durbin R. 2009. 1000 genome project data processing subgroup. The sequence alignment/Map format and SAMtools. Bioinformatics 25:2078–2079. doi:10.1093/bioinformatics/btp352 19505943 PMC2723002

[5] Camacho C, Coulouris G, Avagyan V, Ma N, Papadopoulos J, Bealer K, Madden TL. 2009. BLAST+: architecture and applications. BMC Bioinformatics 10:421. doi:10.1186/1471-2105-10-421 20003500 PMC2803857

[6] Yoon SH, Ha SM, Lim JM, Kwon SJ, Chun J. 2017. A large-scale evaluation of algorithms to calculate average nucleotide identity. Antonie Van Leeuwenhoek 110:1281–1286. doi:10.1007/s10482-017-0844-4 28204908

[7] Seemann T. 2014. Prokka: rapid prokaryotic genome annotation. Bioinformatics 30:2068–2069. doi:10.1093/bioinformatics/btu153 24642063

[8] Huerta-Cepas J, Szklarczyk D, Heller D, Hernández-Plaza A, Forslund SK, Cook H, Mende DR, Letunic I, Rattei T, Jensen LJ, von Mering C, Bork P. 2019. eggNOG 5.0: a hierarchical, functionally and phylogenetically annotated orthology resource based on 5090 organisms and 2502 viruses. Nucleic Acids Res 47:D309–D314. doi:10.1093/nar/gky1085 30418610 PMC6324079

